# Prominent luminescence of silicon-vacancy defects created in bulk silicon carbide p–n junction diodes

**DOI:** 10.1038/s41598-021-81116-8

**Published:** 2021-01-15

**Authors:** Fumiya Nagasawa, Makoto Takamura, Hiroshi Sekiguchi, Yoshinori Miyamae, Yoshiaki Oku, Ken Nakahara

**Affiliations:** Rohm Research and Development Center, ROHM Co., Ltd., Kyoto, Japan

**Keywords:** Lasers, LEDs and light sources, Nanophotonics and plasmonics, Quantum optics, Semiconductors

## Abstract

We investigate fluorescent defect centers in 4H silicon carbide p–n junction diodes fabricated via aluminum-ion implantation into an n-type bulk substrate without the use of an epitaxial growth process. At room temperature, electron-irradiated p–n junction diodes exhibit electroluminescence originating from silicon-vacancy defects. For a diode exposed to an electron dose of $1 \times 10^{18}\,{{\mathrm{cm}}}^{-2}$ at $800\,{{\mathrm{keV}}}$, the electroluminescence intensity of these defects is most prominent within a wavelength range of 400–$1100\,{{\mathrm{nm}}}$. The commonly observed ${{\mathrm{D}}}_1$ emission was sufficiently suppressed in the electroluminescence spectra of all the fabricated diodes, while it was detected in the photoluminescence measurements. The photoluminescence spectra also displayed emission lines from silicon-vacancy defects.

The silicon-vacancy (${{\mathrm{V}}}_{{{\mathrm{Si}}}}$) defect in silicon carbide (SiC) is currently one of the most promising fluorescent defect centers for industrial applications. Applications of this defect have been widely proposed, including magnetic and/or temperature sensors^1–5^, qubits^6,7^, single photon emitters^8^, and microwave emitters^9^. Industrial and academic interest have promoted research on the diamond nitrogen-vacancy center^10^ for several decades. However, the ${{\mathrm{V}}}_{{{\mathrm{Si}}}}$ defect in SiC has several advantages compared to diamond, including the mature process technology and material availability of SiC owing to the recent success of SiC power devices.

From an industrial point of view, the high manufacturing cost of SiC power devices^11^ hinders the industrialization of ${{\mathrm{V}}}_{{{\mathrm{Si}}}}$-based SiC devices. Because SiC epitaxy is one of the most expensive processes involved in manufacturing these devices^12^, a wafer structure without epitaxial layers is preferable. Another crucial issue concerns the excitation method of ${{{\mathrm{V}}}}_{{{\mathrm{Si}}}}$ defects. Optical excitation has been used in a number of reported experiments^2–9^. However, this method requires an additional optical pumping source and fine optical alignment, consequently leading to high costs. Electrical excitation, conversely, is mandatory in practice because it simplifies the system. To the best of our knowledge, no electroluminescence (EL) measurements of ${{{\mathrm{V}}}}_{{{\mathrm{Si}}}}$ defects in bulk SiC have yet been reported. However, several groups have reported EL measurements of color centers in epitaxial SiC films^13–16^. All such results show a two-color spectrum, indicating the inevitable generation of several types of luminescent defects. Multiple-color emissions are not acceptable in practice because of the resulting inefficient excitation of specific color centers. The commonly observed, but always unintentionally introduced, emission band stems from the so-called ${{{\mathrm{D}}}}_1$ center^17^, which is associated with a silicon antisite (${{{\mathrm{Si}}}}_{{{\mathrm{C}}}}$) defect. The suppression of the ${{{\mathrm{D}}}}_1$ emission band in EL spectra is therefore of great importance to realize ${{\mathrm{V}}}_{{{\mathrm{Si}}}}$-functional devices.

In this study, we used a commercially available n-type 4H-SiC substrate, i.e., one that is widely used for power devices, to fabricate p–n junction diode structures. The p-type region was formed via aluminum (Al) ion implantation instead of p-SiC epitaxy. ${{\mathrm{V}}}_{{{\mathrm{Si}}}}$ defects were generated in the SiC substrate via electron irradiation. The luminescence was measured using both electrical and optical excitation methods. The experimental results demonstrate that ${{\mathrm{V}}}_{{{\mathrm{Si}}}}$-originated emission dominates the EL spectra, while other emissions, e.g., ${{{\mathrm{D}}}}_1$ center-related emission, are negligible.

## Results and discussion

**Figure 1:**
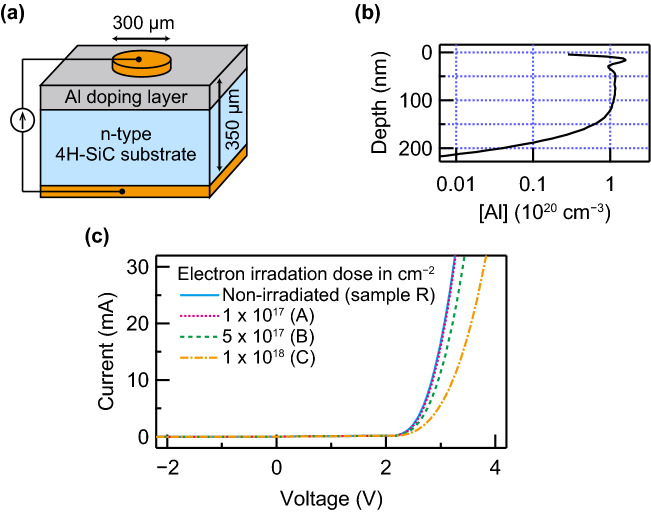
p–n junction diode in a bulk 4H-SiC substrate. (**a**) Schematic of a fabricated p–n junction structure. The nitrogen concentration in the n-type SiC substrate is on the order of $10^{19}\,{{\mathrm{cm}}}^{-3}$. A p-type layer was formed via the multistep implantation of Al ions. (**b**) Depth profile of the Al concentration, [Al]. In the flat-profile region, [Al] exceeds the nitrogen concentration. (**c**) Current–voltage characteristics of the samples with an electron irradiation dose of 0 (sample R), $1 \times 10^{17}$ (A), $5 \times 10^{17}$ (B), and $1 \times 10^{18}\,{{\mathrm{cm}}}^{-2}$ (C). An increase in the electrical resistance is observed for the high-irradiation-dose samples.

Figure 1a shows the sample structure fabricated in this study. A single p–n junction was formed via Al-ion implantation into an n-type 4H-SiC substrate followed by annealing at $1650\,{}^{\circ }{\mathrm{C}}$. A simulated Al concentration^18^ in a doping layer is shown in Fig. 1b. Electron irradiation at the energy of $800\,{{\mathrm{keV}}}$ was performed to introduce intrinsic defects. We prepared four groups of samples with different irradiation doses (sample A: $1 \times 10^{17}\,{{\mathrm{cm}}}^{-2}$, B: $5 \times 10^{17}\,{{\mathrm{cm}}}^{-2}$, C: $1 \times 10^{18}\,{\mathrm{cm}}^{-2}$, and R: non-irradiated reference). The current–voltage characteristics of all the samples are shown in Fig. 1c. A higher electron-irradiation dose increases the electrical resistance. We attribute such an increase to the generation of the non-radiative recombination centers of ${{\mathrm{Z}}}_{1/2}$ and ${\mathrm{EH}}_{6/7}$^19^. The high nitrogen concentration in the substrate prevented the samples from reached a semi-insulating state.

**Figure 2:**
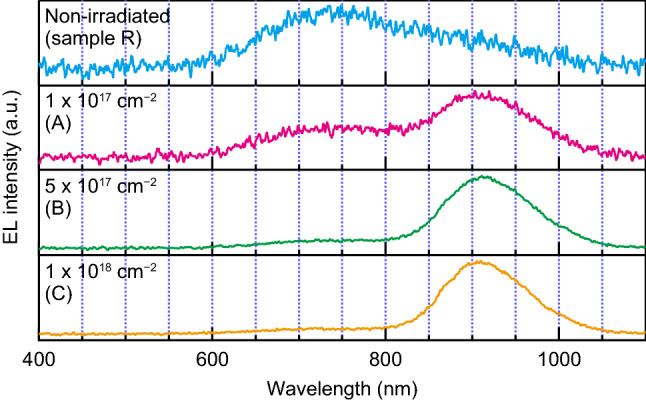
Electroluminescence (EL) spectra at a temperature of $300\,{{\mathrm{K}}}$. All measurements were performed at a bias current of $20\,{{\mathrm{mA}}}$. Labels in each panel indicate the conditions of the electron irradiation dose. Note that the vertical scales are different for each panel. An emission peak around $910\,{{\mathrm{nm}}}$, originating from the silicon-vacancy defects, appears with electron irradiation. This emission dominates the EL spectrum for the electron-irradiated samples (A, B, and C). The broad emission band around $720\,{{\mathrm{nm}}}$ is attributed to the annealing-related defects^20^. ${{{\mathrm{D}}}}_1$ luminescence was not observed.

Figure 2 shows the room-temperature EL spectra at a driving current of $20\,{{\mathrm{mA}}}$. As can be seen in Fig. 2, sample R shows a broad emission band around $720\,{{\mathrm{nm}}}$. In addition to this band, another emission band with a peak wavelength at $910\,{{\mathrm{nm}}}$ appeared for samples A, B, and C. The emission at $910\,{{\mathrm{nm}}}$ is a characteristic of ${{\mathrm{V}}}_{{{\mathrm{Si}}}}$ defects^21^. The ${{\mathrm{V}}}_{{{\mathrm{Si}}}}$-related emission dominates the spectra of samples A, B, and C. In particular, for sample C, it exceeds the other emission peaks by a factor of 11. Conversely, the ${{\mathrm{V}}}_{{{\mathrm{Si}}}}$ emission is negligibly small for sample R. Because the post-implantation annealing temperature of $1650\,{}^{\circ }{{\mathrm{C}}}$ was sufficiently higher than the anneal-out temperature of the ${{\mathrm{V}}}_{{{\mathrm{Si}}}}$ defects, i.e., $750\,{}^{\circ }{{\mathrm{C}}}$^22^, the ${{\mathrm{V}}}_{{{\mathrm{Si}}}}$ concentration introduced by the Al-ion implantation was significantly reduced during the annealing process. Therefore, the ${{\mathrm{V}}}_{{{\mathrm{Si}}}}$ defects in samples A, B, and C were generated purely via the electron irradiation.

**Figure 3:**
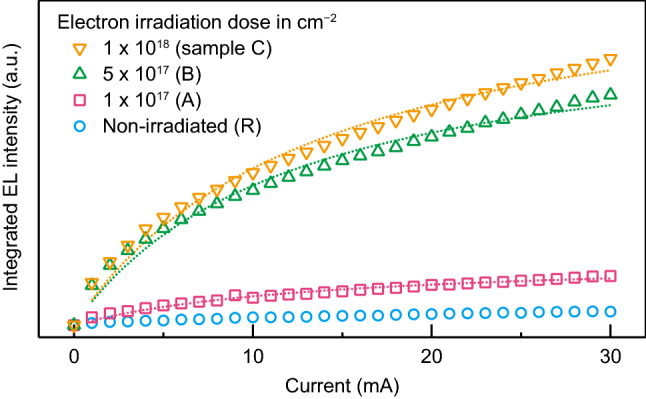
Current dependence of the integrated electroluminescence (EL) intensity for the $910\,{{\mathrm{nm}}}$ emission band. The integration range is fixed to $910 \pm 60\,{{\mathrm{nm}}}$. Saturation of the integrated EL intensity is observed for the irradiated samples (A, B, and C). Dotted lines represent fits using the saturation-curve equation^23^.

Figure 3 shows the integrated intensity of the ${\mathrm{V}}_{{{\mathrm{Si}}}}$ emission as a function of the injection current *I*. Saturated behavior for large *I* is observed for samples A, B, and C. The fit of the equation^23^ $P \propto \left( 1 + I_{0} / I \right) ^{-1}$, where *P* is the EL integrated intensity and $I_0$ is the saturation current, yields $I_{0} = 13.7\,{{\mathrm{mA}}}$ for sample C. This value is larger than the value of $I_0 = 5.3\,{{\mathrm{mA}}}$ for the nitrogen-vacancy-related EL of diamond^24^ but comparable to the value of $I_0 = 10\,{{\mathrm{mA}}}$ for the ${{\mathrm{V}}}_{{{\mathrm{Si}}}}$-related EL of 6H-SiC^13^.

We now turn to the $720\,{{\mathrm{nm}}}$ band observed in the EL spectra in Fig. 2. The origin of this band seems neither carbon antisite–vacancy pair (${{{\mathrm{C}}}}_{{{\mathrm{Si}}}}{{\mathrm{V}}}_{{{\mathrm{C}}}}$) defects^25,26^ nor ${{{\mathrm{D}}}}_1$ defects, but annealing-related defects^20^. The ${{\mathrm{C}}}_{{{\mathrm{Si}}}}{{\mathrm{V}}}_{{{\mathrm{C}}}}$ defect is reported to completely disappear with high-temperature annealing around $1100\,{}^{\circ }{{\mathrm{C}}}$^26^ which is much lower than the employed annealing temperature. In addition, their zero-phonon lines, AB lines^25,26^, were not detected in the photoluminescence (PL) measurements at $10\,{\mathrm{K}}$ (see Supplementary Fig. S1a online). Regarding the ${{{\mathrm{D}}}}_1$ defects, the annealing out of them should not occur in our experiments because these defects are thermally stable up to $1700\,{}^{\circ }{{\mathrm{C}}}$^27^. However, although the ${{{\mathrm{D}}}}_1$ emission intensity should increase with electron irradiation^28^, Supplementary Fig. S2 shows that their EL intensity is not affected by electron irradiation. Thus, there is no good reason to attribute the $720\,{{\mathrm{nm}}}$ band to ${{\mathrm{C}}}_{{{\mathrm{Si}}}}{{\mathrm{V}}}_{{{\mathrm{C}}}}$ or ${{{\mathrm{D}}}}_1$ defects. The annealing-related defects may be the origin of this band, however, their varied emission wavelength for each defect^20^ makes further identification difficult.

In the following, we discuss plausible mechanisms underlying the absence of ${{{\mathrm{D}}}}_1$-originated EL. Unlike the EL measurements, the ${{{\mathrm{D}}}}_1$ emission was observed in the PL measurements for samples R and C even at room temperature, and was not observed in a bare SiC substrate (see Supplementary Fig. S1b online). Therefore, ${{{\mathrm{D}}}}_1$ defects were introduced at least to the penetration depth of excitation light from the top-surface via Al-ion implantation and electron irradiation. Based on the above results, the deteriorated crystal quality in the vicinity of the p–n junction by the high-fluence implantation of Al ions may be the mechanism of the smeared ${{{\mathrm{D}}}}_1$ band in all of the EL spectra. It has been established that variations in the stacking faults of SiC are sensitive to the wavelength of the ${{\mathrm{D}}}_1$-emission line^14^; therefore, this interpretation is reasonable. According to this mechanism, our EL results suggest that ${{{\mathrm{D}}}}_1$ defects are more sensitive to the crystal quality than ${{\mathrm{V}}}_{{{\mathrm{Si}}}}$ because ${{\mathrm{V}}}_{{\mathrm{Si}}}$-related EL can still be observed in the electron-irradiated, i.e., additional defect-introduced, samples. Also, defect diffusion is another possible mechanism. The depth profile of Al-implanted 4H-SiC, as detected by cathode luminescence^29^, revealed that high-temperature annealing induces the diffusion of ${{{\mathrm{D}}}}_1$ defects from the surface-implanted area, with a diffusion length of up to several micrometers, while the implanted Al atoms remain in the vicinity of the surface. The low concentration of ${{{\mathrm{D}}}}_1$ defects in the p–n junction layer then would result in the absence of ${{{\mathrm{D}}}}_1$-related EL. This mechanism, however, leaves an open question as to why the ${{\mathrm{D}}}_1$-emission band does not appear after electron irradiation (see Supplementary Fig. S2 online); electron irradiation should generate the ${{{\mathrm{D}}}}_1$ defects^28^. Lastly, note, thermal activation of the defect-bound electrons in the conduction band^13^ due to current injection is not the mechanism of the absence of ${{{\mathrm{D}}}}_1$ EL, because the comparison of the EL spectra between the smallest ($I = 1\,{{\mathrm{mA}}}$) and largest ($30\,{{\mathrm{mA}}}$) currents revealed that there was no significant difference in the shape of EL spectra and the ${{{\mathrm{D}}}}_1$ EL was not detected for the both currents (see Supplementary Fig. 3 online).

**Figure 4:**
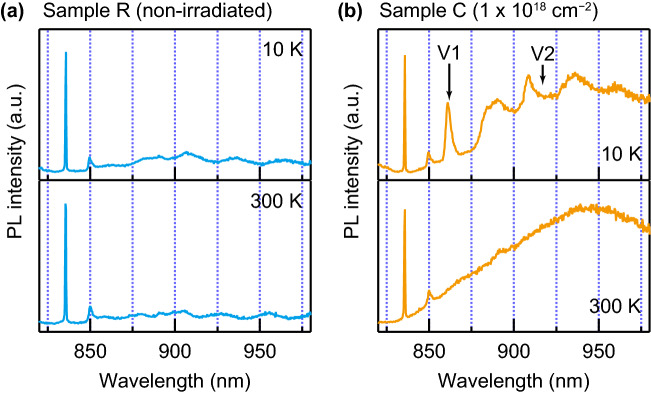
Photoluminescence (PL) spectra with a 785-nm excitation laser at room temperature. The excitation energy is $3.7\,{{\mathrm{mW}}}$ at the top surface of the samples. The PL spectrum for the (a) non-irradiated and (b) $1 \times 10^{18}\,{\mathrm{cm}}^{-2}$-irradiated samples. Measurements were performed at temperatures of $10\,{{\mathrm{K}}}$ (upper panel) and $300\,{{\mathrm{K}}}$ (lower panel). The ${{\mathrm{V}}}_1$ zero-phonon line of the silicon-vacancy defects is clearly seen at $10\,{{\mathrm{K}}}$ for the irradiated sample. The emission lines at $836\,{{\mathrm{nm}}}$ and $850\,{{\mathrm{nm}}}$ originate from the Raman scattering of 4H-SiC^30^.

The PL spectra of samples R and C with a 785-nm excitation laser are shown in Fig. 4. Except for the Raman scattering peaks of 4H-SiC at $836\,{{\mathrm{nm}}}$ and $850\,{{\mathrm{nm}}}$^30^, as shown in the upper panel of Fig. 4a, no significant emission peaks were observed for sample R at $10\,{{\mathrm{K}}}$. As can be seen in the lower panel of Fig. 4a, the PL spectrum at $300\,{{\mathrm{K}}}$ does not show a large difference. These results indicate the absence of ${{\mathrm{V}}}_{{{\mathrm{Si}}}}$ defects in sample R. Conversely, as shown in the upper panel of Fig. 4b, the 862-nm emission line, labeled V1, appears in the PL spectrum of sample C at $10\,{{\mathrm{K}}}$. This emission line originates from the ${{\mathrm{V}}}_1$ zero-phonon line of the ${{\mathrm{V}}}_{{{\mathrm{Si}}}}$ defects in 4H-SiC^31^. The ${{\mathrm{V}}}_2$ emission line at $917\,{\mathrm{nm}}$ was too weak to be assigned compared to the large phonon sideband (PSB), which corresponds to the large Huang–Rhys (HR) factor (the small Debye–Waller factor^32^). Note, ${{\mathrm{\mathrm V'}}}_{1}$ line, which is associated with a second excited state of the ${{\mathrm{V}}}_{{{\mathrm{Si}}}}$ defect^33^, was not detected possibly due to a low measurement temperature^31^. The 300-K PL spectrum of sample C in the lower panel of Fig. 4b shows a broad PSB originating from the ${\mathrm{V}}_{{{\mathrm{Si}}}}$ defects with its peak wavelength around $940\,{\mathrm{nm}}$, which is slightly longer than the typical wavelength for the ${{\mathrm{V}}}_{{{\mathrm{Si}}}}$ PL. However, the PSB peak around $950\,{\mathrm{nm}}$ was observed with the experimental setup similar to ours^34^, and is reasonable for the large HR factor. The discrepancy of the peak wavelength of the ${{\mathrm{V}}}_{{\mathrm{Si}}}$-related PSB between the EL and PL spectra is perhaps due to the different local crystalline structures under detection. Therefore, both the EL and PL measurements substantiate the feasibility of efficient ${{\mathrm{V}}}_{{{\mathrm{Si}}}}$ luminescence emitted from a bulk SiC substrate with no epitaxial layers.

## Conclusion

In conclusion, we successfully fabricated p–n junction diodes including ${{\mathrm{V}}}_{{{\mathrm{Si}}}}$ defects on a bulk 4H-SiC substrate without epitaxial layers. The PL spectra demonstrated the formation of ${{\mathrm{V}}}_{{{\mathrm{Si}}}}$ defects. The EL measurements revealed that the luminescence intensity of ${{\mathrm{V}}}_{{{\mathrm{Si}}}}$ was most prominent within the measured wavelength range. Further, the commonly observed ${{{\mathrm{D}}}}_1$ emission was sufficiently suppressed in the EL spectra, while it was detected in the PL measurements. The absence of the ${{{\mathrm{D}}}}_1$ emission in the EL spectra suggests that the deteriorated crystal quality due to ion implantation affects its luminescence. Our approach provides a foundation for novel applications of ${{\mathrm{V}}}_{{{\mathrm{Si}}}}$ defects in SiC using simple manufacturing processes.

## Methods

We employed an n-type 4H-SiC substrate with a nitrogen concentration on the order of $10^{19}\,{{\mathrm{cm}}}^{-3}$ purchased from SiCrystal AG. Multi-energy Al-ion implantation was performed at a substrate temperature of $500\,{}^{\circ }{{\mathrm{C}}}$ to form a p-type region on the top-surface of the substrate. The fluence and energy values of the Al implantation were $2.0 \times 10^{14}$, $2.0 \times 10^{14}$, $2.5 \times 10^{14}$, $2.5 \times 10^{14}$, and $8.0 \times 10^{14}\,{{\mathrm{cm}}}^{-2}$ at 10, 30, 50, 70, and $100\,{\mathrm{keV}}$, respectively. According to an SRIM-code simulation^18^, the Al atoms should be flatly distributed from the surface to a depth of $130\,{{\mathrm{nm}}}$ with an Al concentration of $1 \times 10^{20}\,{{\mathrm{cm}}}^{-3}$. Post-implantation annealing was performed at $1650\,{}^{\circ }{{\mathrm{C}}}$ for 30 min in an argon atmosphere. Subsequent to annealing, the substrate was diced into chips. These chips were divided into four groups. The first three groups were irradiated with 800-keV electrons at doses of $1 \times 10^{17}$ (sample A), $5 \times 10^{17}$ (sample B), and $1 \times 10^{18}\,{{\mathrm{cm}}}^{-2}$ (sample C). The remaining group (sample R) was not irradiated by electrons and served as a reference. Finally, Ti/Ni/Au Schottky contact metal was deposited on the top and bottom surfaces of the chips via electron-beam evaporation. The top-surface electrodes were circularly shaped with a diameter of $300\,\upmu {\mathrm{m}}$.

## Supplementary Information

Supplementary Information.

## Data Availability

The data that support the findings of this study are available from the corresponding author on reasonable request.
