# Supplementary material for: Prominent luminescence of silicon-vacancy defects created in bulk silicon carbide p–n junction diodes
Source: Sci Rep. 2021 Jan 15;11:1497. doi: 10.1038/s41598-021-81116-8 (PMC7810994; doi:10.1038/s41598-021-81116-8)
Supplement: Supplementary file 1 — Supplementary Information. [file 41598_2021_81116_MOESM1_ESM.pdf]

Supplementary information

Prominent luminescence of silicon-vacancy defects created in bulk silicon carbide p–n junction diodes

Fumiya Nagasawa*, Makoto Takamura, Hiroshi Sekiguchi, Yoshinori Miyamae, Yoshiaki Oku, and Ken Nakahara

Rohm Research & Development Center, ROHM Co., Ltd., Kyoto, Japan
*fumiya.nagasawa@dsn.rohm.co.jp

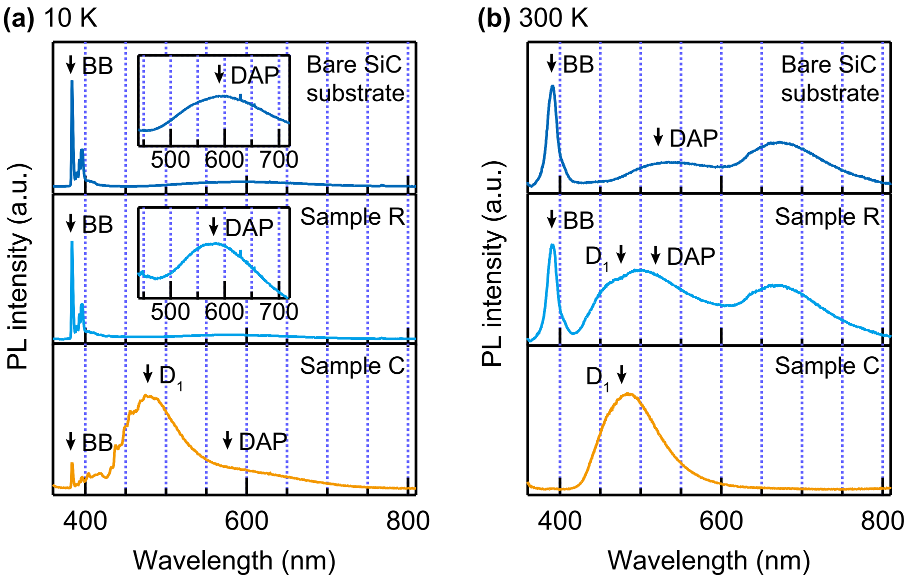

Supplementary Figure S1. Photoluminescence (PL) spectra with a 325-nm excitation laser at (a) 10 K and (b) 300 K. The excitation energy is 2.0 mW at the top surface of the samples. For a bare substrate and sample R, the donor–acceptor pair (DAP) luminescence arising from nitrogen–boron pairs[1] is observed to range between 500 nm and 600 nm, depending on the temperature. The D_1 emission appears around 480 nm for samples R (non-irradiated) and C (irradiation dose of $1 \times 10^{18}\,\mathrm{cm}^{-2}$). A band-to-band (BB) emission of 4H-SiC is observed in all the measurements except for sample C at 300 K. In the wavelength range above 800 nm, the PL signal was dominated by the strong interference. The zero-phonon lines of the carbon antisite–vacancy pair[2] were not detected. The DAP luminescence originating from nitrogen–aluminum pairs, the emission wavelength of which is around 400 nm[1], is weak in our samples because the aluminum-ion implantation region is shallow compared to the excitation-light penetration depth.

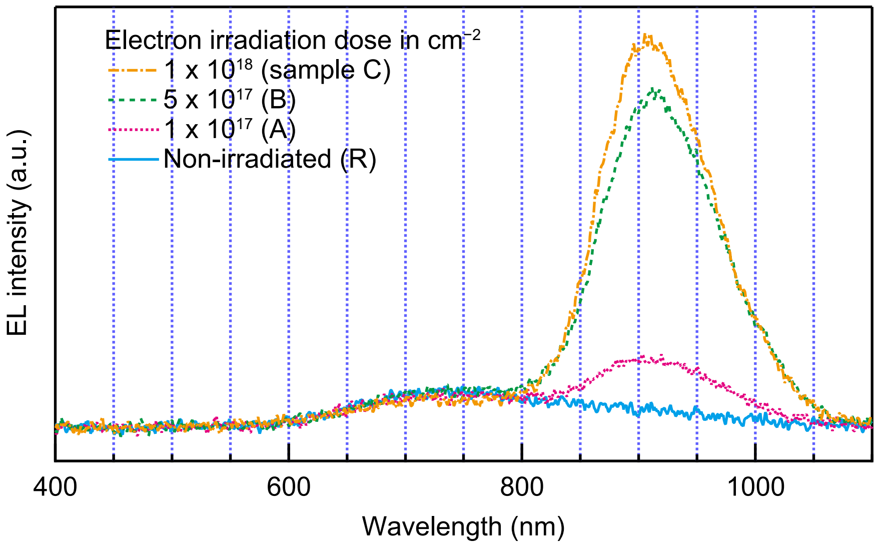

Supplementary Figure S2. Comparison of the relative intensity of electroluminescence (EL) spectra. All measurements were performed at a bias current of 20 mA. The peak intensity of the 720 nm band does not vary significantly between all the samples; therefore, electron irradiation does not affect this band. Conversely, the peak intensity of the 910 nm band, originating from the silicon-vacancy defects, increases with electron irradiation.

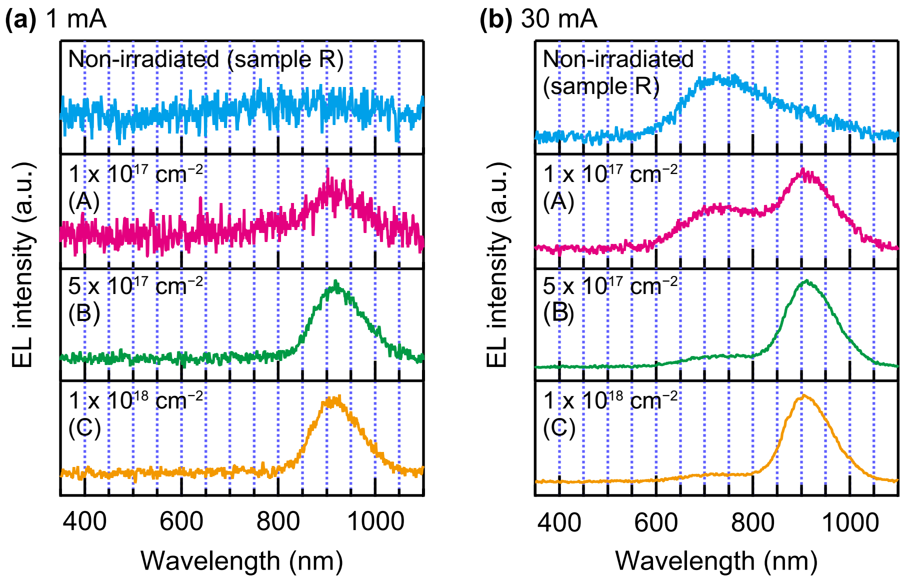

Supplementary Figure S3. Current dependence of the electroluminescence (EL) spectra at room temperature. The measurements were performed at bias currents of (a) 1 mA and (b) 30 mA. Labels in each panel indicate the conditions of the electron irradiation dose. Note that the vertical scales are different for each panel. D_1 luminescence was not observed for the both bias currents.

References

1. Liu, X. *et al.* Donor-acceptor-pair emission in fluorescent 4H-SiC grown by PVT method. *AIP Adv.* **5**, 047133, DOI: 10.1063/1.4919012 (2015).

2. Castelletto, S. *et al.* A silicon carbide room-temperature single-photon source. *Nat. Mater.* **13**, 151–156, DOI: 10.1038/nmat3806 (2014).
